# First Confirmed Occurrence of Ciguatera Poisoning in the UK from Imported Pinjalo Snapper (*Pinjalo pinjalo*)

**DOI:** 10.3390/md23020067

**Published:** 2025-02-06

**Authors:** Andrew D. Turner, Benjamin H. Maskrey, David Stone, Elizabeth M. Mudge, Alison Robertson

**Affiliations:** 1 Centre for Environment Fisheries and Aquaculture Science (Cefas), Barrack Road, Weymouth DT4 8UB, UK; ben.maskrey@cefas.gov.uk (B.H.M.); david.stone@cefas.gov.uk (D.S.); 2 Biotoxin Metrology, National Research Council Canada, Halifax, NS B3H 3Z1, Canada; elizabeth.mudge@nrc-cnrc.gc.ca; 3 Dauphin Island Sea Lab, 101 Bienville Blvd, Dauphin Island, AL 36528, USA; arobertson@disl.org; 4 Department of Marine Sciences, University of South Alabama, University Avenue N., Mobile, AL 36688, USA

**Keywords:** ciguatera, ciguatoxins, fish toxins, snapper, *Pinjalo pinjalo*, Europe, United Kingdom

## Abstract

Three people in England consumed fish steaks labeled as Red Snapper (*Lutjanus bohar*) originating from the Indian Ocean. Within 12 h, all three experienced sickness including nausea, vomiting, diarrhea, as well as myalgia and paresthesia. Three steaks from a single package of fish obtained from a grocery store were consumed, leaving one uneaten, which was submitted for analysis. Cytotoxicity testing via the mouse neuroblastoma assay confirmed the presence of sodium channel specific activity consistent with a ciguatoxin standard, and the levels detected were above established guidance limits for safe consumption. Chemical detection using liquid chromatography coupled with high-resolution mass spectrometry of both intact toxins and periodate oxidation products was used to confirm the presence of chromatographic peaks consistent with tri- and di-hydroxylated Pacific ciguatoxin 3C congeners. Taking the shared medical symptoms of patients, the recent dietary history, and the known potential for ciguatera poisoning to occur in snapper species, the subsequent evidence for CTX-like activity and CTXs in the same fish sample provides very strong evidence that the fish steaks consumed were similarly contaminated with CTXs. Furthermore, given the levels reported, such toxicity would be expected to cause intoxication in humans. Fish species identification based on DNA barcoding confirmed that the fish products were mislabeled, with the tissues instead being the *Pinjalo* snapper, *Pinjalo pinjalo*. This is the first confirmed ciguatera poisoning incident in both the UK and from the *Pinjalo* snapper and highlights the need for monitoring of these emerging toxins in reef fish imports to prevent future human intoxication.

## 1. Introduction

**Figure 1:**
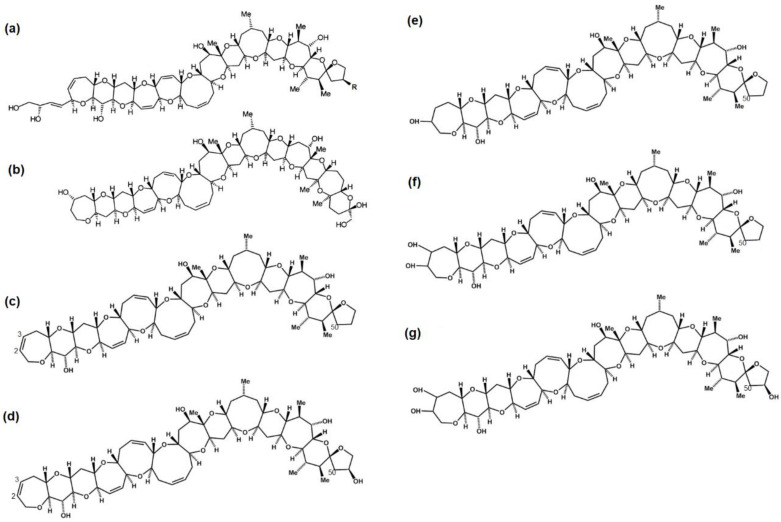
Example chemical structures of Pacific and Caribbean ciguatoxin groups; (**a**) Pacific ciguatoxin 1 (CTX-1B) (R=OH) and Pacific ciguatoxin 3 (54-deoxy-CTX-1B) (R=H) (**b**) Caribbean ciguatoxin-1 (C-CTX-1) (**c**) CTX-3C (**d**) 51-hydroxy-CTX-3C (**e**) 2-hydroxy-CTX-3C (**f**) 2,3-dihydroxy-CTX-3C (**g**) 2,3,51-trihydroxy-CTX-3C.

It is estimated that 10,000 to 50,000 people are affected by ciguatera poisoning each year, although it is believed that only 2–10% of cases are reported [1,2,3,4]. Ciguatoxin-group toxins (CTXs) are a diverse family of toxins with up to ~40 congeners of ladder-like polyether compounds containing 13–14 ether rings reported with varying levels of conformation. CTXs have been reported in reef-associated organisms in the Pacific, Caribbean and Indian Oceans and are traditionally classified as (P), (C) or (I) CTX-group toxins, respectively [5] (Figure 1). They occur through the enzymatic oxidation and biotransformation of precursor molecules produced by the benthic dinoflagellates *Gambierdiscus* spp. and *Fukuyoa* spp., which typically thrive on biotic and artificial substrates in shallow marine environments (<30 m) [6,7,8,9,10,11,12,13]. Ciguatera is often associated with the consumption of large predatory reef fish [5,9,14,15,16,17,18,19], although invertebrate vectors have also been reported [20,21]. The toxins have been detected in a variety of fish genera and species spanning multiple trophic levels across the world [5,14,16,17]. Though stability studies are currently lacking, CTXs are thought to be heat- and low-temperature-stable based on a few reports, so it has been assumed that they cannot be destroyed through routine cooking or freezing [22,23].

Ingestion of the toxins cause the syndrome ciguatera, which is characterized by a complex range of clinical signs, including gastrointestinal, neurological and cardiovascular effects, and death can occur from cardiac or respiratory failure in rare cases [24,25,26]. The voltage-gated sodium channel has been identified as the primary molecular target for the toxins, with toxin binding causing prolonged opening of the channel and influx of sodium to the cell. The situation is further complicated by the variability in symptoms experienced depending on the region, consumption patterns, and culturally specific behaviors of consumers [4,27,28,29,30,31,32,33,34,35,36]. Differences in clinical presentations, severity, and duration of illness have been recorded, even among consumers of the same fish [5,37]. The toxins are extremely potent, with LD_50_ in mice (I.P.) equivalent to as low as 0.9 µg/kg depending on the congener [38]. Whilst no regulatory limits or methods of analysis are set currently within Europe or the United States, European Commission (EC) regulations do require that checks are made to ensure that fishery products do not contain biotoxins such as CTX [39]. The USA FDA has proposed guidance levels of <0.1 µg/kg for C-CTX-1 and <0.01 µg/kg for CTX-1B, but these are not regulations, and the guidance only suggests the avoidance of highly endemic regions and/or high-risk species.

In recent years, there have been increasing numbers of reports describing evidence for the presence of CTXs and/or the causative dinoflagellates within European waters [4,32,40,41,42,43,44,45,46,47]. *Gambierdiscus* spp. have been detected as far east as Crete in 2003 [41] and as far west as the Canary Islands and Madeira in 2008 [42,48,49]. In the Atlantic, human intoxications have been linked with consumption of Amberjack (*Seriola rivoliana*) and *S. dumerili* and *S. fasciata*, in the Canary Islands and Madeira, respectively [40,50,51]. CTXs were also reported in *S. fasciata* from the Selvegen Islands, Portugal, and implicated in poisonings in the Canary Islands [43]. Other fish species implicated in the region include Island grouper (*Mycteroperca fusca*), Dusky grouper (*Epinephelus marginatus*), Barred hogfish (*Bodianus scrofa*) and grey triggerfish (*Balistes capriscus*) [47]. More recently, LC-HRMS has been used to confirm the presence of C-CTX analogues including hydroxy-, didehydro- and methoxy-metabolites [46]. Overall, the majority of reports of ciguatera occurring in Europe predominantly relate to travel to endemic countries bordering known hot spots of the syndrome, where the causative algae are known to occur [52,53] or from imported fish (*L. bohar*) from Indo-Pacific waters such as have been reported in Germany and France [18,54,55,56,57].

**Figure 2:**
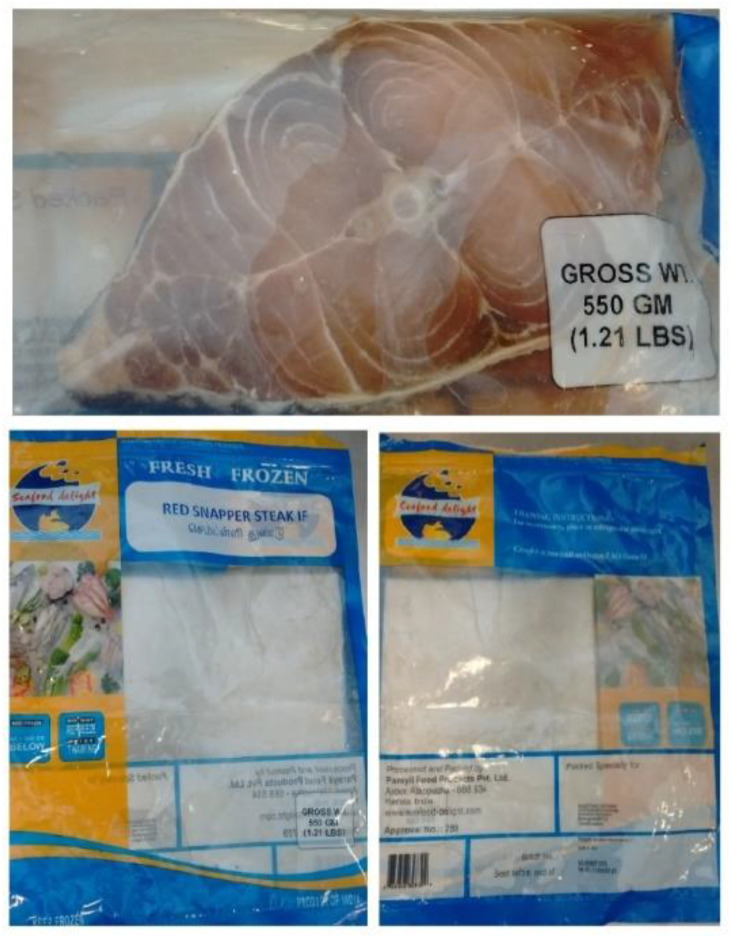
Images of implicated fish steak and packaging.

In June 2017, a suspected ciguatera incident occurred in England associated with the consumption of fish marketed as red snapper (*L. bohar*) steaks. Three people were affected from the same family, all of whom consumed separate steaks from the same product package (Figure 2). Investigations revealed that the fish were harvested and processed in India and imported to the UK. The Food Standards Agency of the UK were made aware of the incident (reference number N_3861), and a product withdrawal was put in place for the brand associated with the intoxication. Two of the three family members were admitted to hospital with symptoms of diarrhea and vomiting, followed by paresthesia and shortness of breath. The reporting doctor provided a presumptive diagnosis in the absence of analytical confirmation that ciguatera was the likely cause of illness.

To date, few reports of ciguatera originate from India or from fish harvested from the Indian Ocean, with a recent review highlighting the lack of CTX or CTX-producing microalgae-related data from the region [18]. Human poisonings have occurred mainly in and around coastal island regions and reef habitat of the SW Indian Ocean, incorporating Comoros, Mayotte, La Reunion, Mauritius, Rodrigues and Seychelles, with a few others in the north, specifically southern India [18] and Malaysia [58]. From the reported incidents, however, some include fatal intoxications following consumption of shark tissues from Madagascar [4,59,60,61,62,63], as well as poisonings from reef fish in Reunion Island [64] and in mainland India [65,66]. Reef fish from the genus Lutjanus (snapper) were assessed for CTX presence by [33,34], who demonstrated CTX-like in vivo biological and sodium-channel activity, and CTXs by LC-MS, from two separate snapper species.

Following the reporting of the event in the U.K., one uneaten fish steak from the implicated batch was sent to the Centre for the Environment, Fisheries and Aquaculture science (Cefas) for assessment. The fish steak was subsequently analyzed for the presence of CTXs using multiple methods. This paper reports the subsequent investigations conducted to establish the cause of the poisonings following the consumption of the imported fish steaks in the U.K. The protocol adopted followed optimized strategies to those previously reported by the United States Food and Drug Administration (US FDA) for suspected ciguatera incidents, specifically involving fish species identification through DNA barcoding, semi-quantitation of CTX activity through use of an in vitro mouse neuroblastoma cell proliferation assay (N2a-MTT), and molecular confirmation of toxin presence with liquid chromatography and mass spectrometry [5,10,17,29,45,67,68,69,70,71,72].

## 2. Results

### 2.1. Fish Speciation

Three steaks from a packet of four were consumed, leaving one uneaten in frozen storage, which was subsequently submitted for DNA barcoding. Fish tissue samples were processed for DNA extraction and barcoding for species identification using partial cytochrome oxidase I gene sequence analysis [73,74,75,76]. The full 536 nucleotide sequence generated from the amplified product shared 100% nucleotide identity with nine partial COI sequences submitted to GenBank for Pinjalo snapper (*Pinjalo pinjalo*) including accessions MT076707, KX060042, OQ385992 and KP856805. The 536 nucleotide sequence shared <86.2% nucleotide identity to the partial COI sequences available for *Lutjanus bohar* on GenBank.

### 2.2. N2a-MTT Cytotoxicity Assay

**Figure 3:**
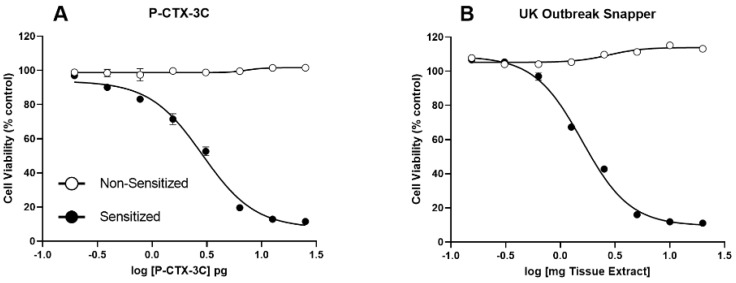
Representative dose-response curves from the N2a-MTT assays performed during this study. N2a cells sensitized with ouabain (O) and veratrine (V) in addition to standard or sample extract dilutions (solid black circles) and non-sensitized cells that received no additional dosing with OV (open circles). The response observed for (**A**) commercially available CTX-3C standard (Wako chemicals); and (**B**) extracts of the UK Snapper (*P. pinjalo*) are shown. Triplicate values and the standard error of the mean are plotted in all cases; however, in some instances, the error bars are obscured by the symbols.

The fish extracts showed positive CTX-like activity by the N2a-MTT assay. A dose-dependent loss in cell viability of N2a cells was observed following treatment with the snapper extract in the presence of ouabain and veratrine, indicating sodium channel-specific activity consistent with a CTX-3C standard (Figure 3). Cell viability was not affected in the non-sensitized cells, indicating that no other toxin class capable of affecting neuroblastoma cells was likely present in the snapper sample extracts. Data were highly reproducible across replicate assays performed on the same date (<5% CV) and across different days with different cell passages (<12% CV). Quantification based on the IC_50_ comparison between the sample and CTX-3C standard curves revealed a concentration of 1.81 µg CTX-3C eq./kg tissue, though the toxicity equivalence between CTX-3C and the causative toxins in our samples could not be determined and was outside the scope of this study since purified analytes would be required and were not available.

### 2.3. Liquid Chromatography-High Resolution Mass Spectrometry (LC-HRMS)

#### 2.3.1. Full Scan

**Figure 4:**
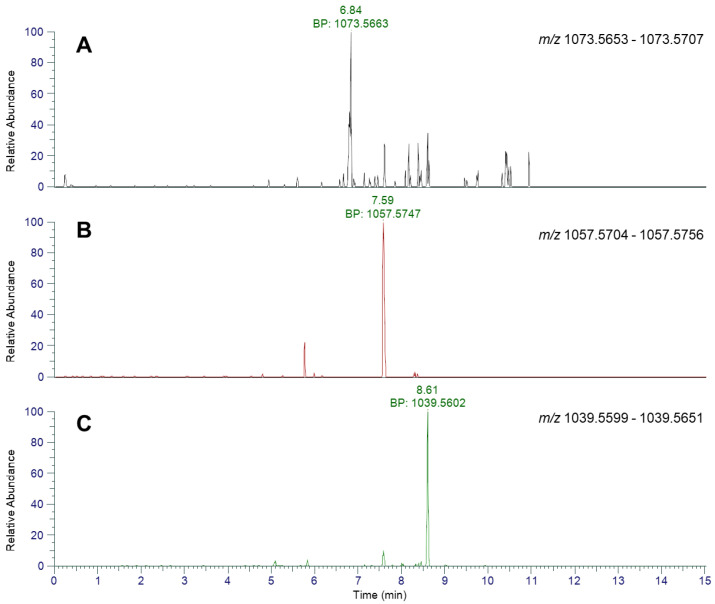
UHPLC-HRMS analysis of hydroxylated CTX-3C analogues in SPE-cleaned snapper tissue extracts, showing base peak chromatograms of (**A**) [M + H]^+^ of 2,3,51-trihydroxy-CTX-3C, (**B**) [M + H]^+^ of 2,3-dihydroxy-CTX-3C, (**C**) [M + H]^+^ of 51-hydroxy-CTX-3C.

Full-scan analysis of the SPE-cleaned snapper tissue extract by UHPLC-HRMS revealed the presence of distinct chromatographic peaks eluting at 6.84, 7.59 and 8.61 min consistent with the masses of tri-, di- and mono-hydroxylated CTX-3C, respectively (Figure 4 and Table 1). Furthermore, the Na^+^ adduct for 2,3,51-trihydroxy-CTX-3C was also obtained (*m*/*z* 1095.5499) with an identical chromatographic retention time to the [M + H]^+^ ion at 6.84 min. The presence of a small peak at 7.59 min at *m*/*z* consistent with the mono- and di- hydroxy CTX-3C is believed to be due to loss of H_2_O within the electrospray source. Interrogation of the data for the presence of ions consistent with the exact masses of CTX-3C and other CTX-3C group congeners identified potential low levels peaks with ions at *m*/*z* 1059.5887 (5.7 min), 1055.5574 (5.25 min) and 1041.5781 (5.83 min) consistent with masses for A-*seco*-51-hydroxy-CTX-3C, 51-hydroxy-2-oxo-CTX-3C and 2-hydroxy-CTX-3C, respectively. No fragmentation of parent ions was performed. No peaks were detected with exact masses consistent to CTX-4A group and the C-CTX group analogues. No NH_4_^+^ adducts were observed for any of the hydroxylated CTX-3C congeners. These peaks were cross referenced against the bulk seized material and previously reported hydroxylated CTX-3C analogues in contaminated tissues from poisonings associated with *L. bohar* from Vietnam [57] (Supplementary Information). No C/I-CTXs were detected.

Analysis of a sub-sample of the fish tissue was extracted and analyzed for paralytic shellfish poisoning (PSP) toxins and tetrodotoxin (TTX), with results showing no detectable levels of these toxins.

#### 2.3.2. Reaction with Periodate

**Figure 5:**
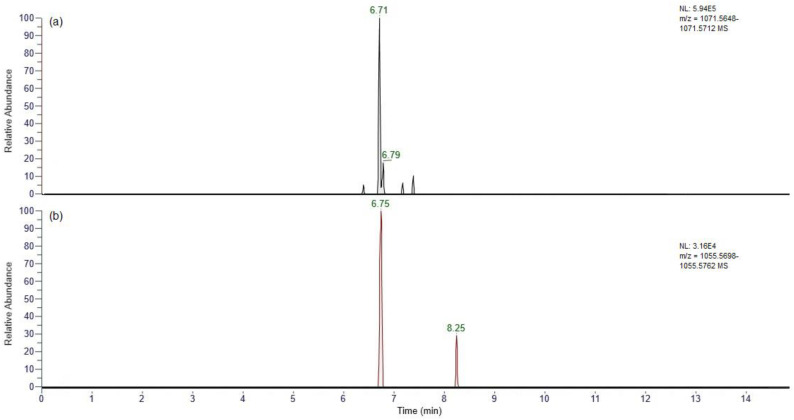
UHPLC-HRMS analysis of periodate oxidation products of (**a**) [M + H]^+^ of 2,3,51-trihydroxy-CTX-3C, (**b**) 2,3-dihydroxy-CTX-3C.

Periodate was used here for the oxidative cleavage of 1,2-diols as demonstrated previously by the oxidation of C-CTXs [77]. Periodate oxidations were applied to 20 µL aliquots of the SPE-cleaned fish extracts, looking for the oxidation products of the hydroxylated CTX-3C analogues. Given the expected carbon bond cleavage between the 2,3 positions on the di- and tri-hydroxylated CTX-3C molecules, reaction products were anticipated to result in the loss of 2H from the original congeners following the formation of two carbonyl moieties (Supplementary Materials, Figure S2). UHPLC-HRMS confirmed the presence of chromatographic peaks eluting at 6.71 min and 6.75 min for the reaction products of 2,3,51-trihydroxy-CTX-3C (*m*/*z* 1071.5680) and 2,3-dihydroxy-CTX-3C (*m*/*z* 1055.5730), respectively, with a mass error of <2 ppm (Figure 5).

### 2.4. Medical Observations

Three female patients were assessed in hospital, with two being held under observation for two days (patients 1 and 2). Patient 3 was observed on day 1, before discharge, and a follow-up hospital visit on day 2. The ages of the three patients were 20, 45 and 11, respectively. The two older females presented similar complaints, including vomiting, diarrhea, myalgia (muscle pain), shortness of breath and paresthesia (which included skin pain, itching, numbness, as well as pins and needles). The third younger patient also experienced bone pain in the right shin, some reduced power in the legs, and diarrhea. Intravenous fluids were administered to patients 1 and 2 but no further treatment was provided. Blood analyses for full blood count, kidney function, i.e., urea and electrolytes, and liver function tests were all considered in the normal range. No neurological anomalies were noted in patient 3. Pain relief medications (codeine, paracetamol, ibuprofen) were provided for patients 1 and 2 following hospital discharge and patient 2 was also provided with antihistamines. Table 2 summarizes the medical reports from each of the three patients. Before any testing commenced, these cases were determined by clinicians to be associated with ciguatera poisoning due to the recent consumption of tropical fish and symptoms deemed consistent with ciguatera.

## 3. Discussion

### 3.1. Location

**Figure 6:**
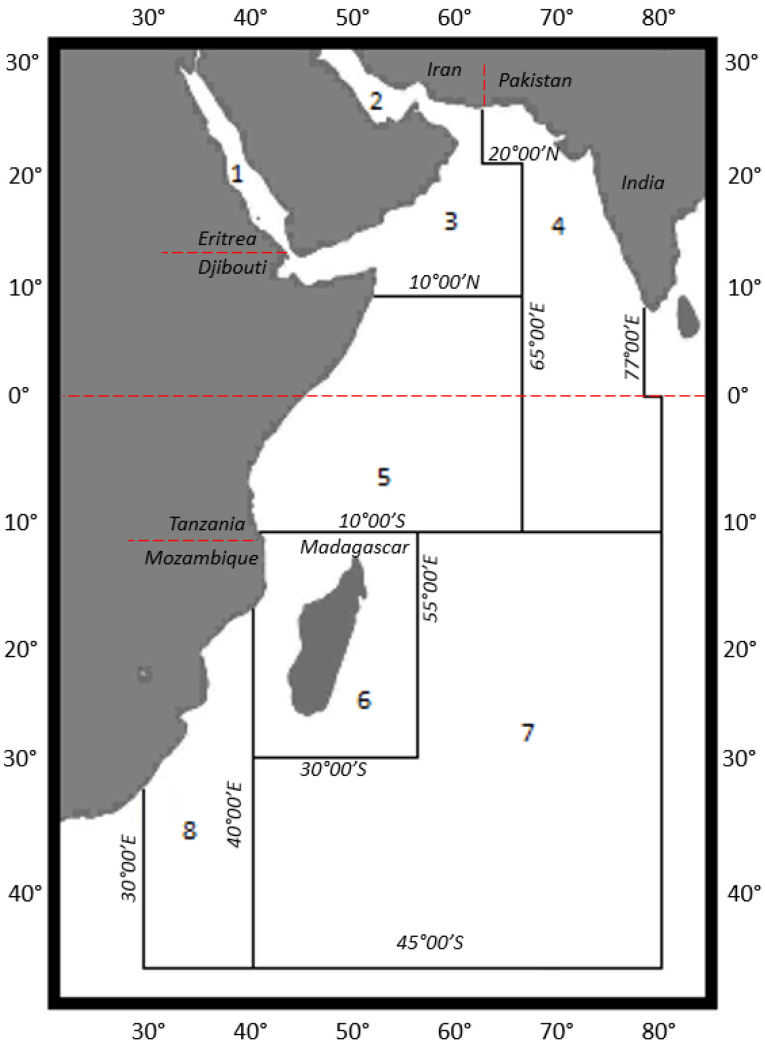
Map illustrating the eight sub-areas defined within FAO Major Fishing Area 51: (1) Red Sea; (2) Gulf; (3) Western Arabian Sea; (4) Eastern Arabian Sea; (5) Somalia, Kenya and Tanzania; (6) Madagascar and Mozambique Channel; (7) Oceanic; (8) Mozambique [78].

Three females experienced sickness, including nausea, vomiting, diarrhea, myalgia, and paresthesia, following the consumption of snapper steaks. The fish had been imported to the UK through a Midlands-based food distributor. The original source of the fish was the Indian Ocean, FAO area 51, with the fish processed in a food production and packaging facility in Alappuzha, Kerala, southern India prior to export. No information was made available concerning the origin of the fish by the food processors upon repeated request. FAO major fishing area 51 is situated in the western Indian Ocean, extending from the Suez Canal and the Iranian Gulf in the north (latitude 30° North) down to the most southerly extent of latitude 45° South. The zone is split into eight sub-areas, incorporating the Red Sea, the Gulf, W. Arabian Sea, E. Arabian Sea and Laccadives, Somalia, Kenya and Tanzania, Madagascar and Mozambique Channel, Oceanic and Mozambique (Figure 6). Consequently, without any further information on the location of harvest/origin of the snapper, it is clear that the implicated samples may have originated from anywhere within a large geographical range [78]. The origin within this zone is therefore largely unknown, but expected to relate to reef-associated areas in warmer waters.

Ciguatera is frequently associated with poisoning incidents in coastal areas and islands of the Pacific and Caribbean Oceans with relatively few poisonings recorded in synonymous regions of the Indian Ocean [32,79], although earliest reports from this region date back to the 17th century [80]. Previous severe outbreaks in Madagascar (sub-area six, Figure 5) have been associated with the consumption of sharks, resulting previously in high fatality rates of more than 20%, with the apparent differences in clinical symptoms between those cases and classic ciguatera. This has raised the possibility of other toxins, carchatoxins, causing intoxication following consumption of sharks [59,60,61,62], although more recently, I-CTXs have been identified using high-resolution mass spectrometry in a bull shark following another fatal food poisoning in Madagascar [63]. Reef fish CTXs were isolated previously from samples of two red snappers/bass (*Lutjanus bohar*) and two Red Emperors (*Lutjanus sebae*) taken from the Soudan fishing banks approximately 100 miles to the north east of Mauritius, located in the north-west of sub-area seven of FAO zone 51 (Figure 5) [33,34]. Ciguatera has also been reported west of Mauritius in Reunion Island, where 375 notified cases of poisonings were reported between 1986 and 1994, even though the sale of reef fish is banned [64]. It is believed that the majority of the fish captured and eaten in Reunion originated from offshore coral banks north of Mauritius, such as the Soudan, Nazareth and Saya de Malha [64]. Most recently, several reports have originated from mainland India, bordering sub-zone 4, describing human intoxications in SW India following consumption of *L. bohar* [81,82], with poisonings in Europe also occurring following consumption of snappers originating from processing establishments in India [57,65,81,83,84,85]. With ciguatera not being reported in India prior to 2016, there are implications for climate-change-related shifts in benthic dinoflagellate communities and a consequent need to monitor coral reef fish species for CTXs [65].

### 3.2. Fish Species

Following DNA barcoding, the fish species was confirmed as being the *Pinjalo* snapper (*Pinjalo pinjalo*) in contrast to the marketed species of Red Snapper (*L. bohar*). Such mislabeling seems not to be uncommon, given similar issues in Germany where snappers sourced from FAO zone 71 (western central Pacific Ocean) labeled as *L. malbaricus* were tested and found to be *L. bohar* [57], and all of which are red-colored snapper species. The *P. pinjalo* from this study are reef-dwelling carnivores, which are typically trawled to a depth of 100 m but can be found in schools in the open ocean in a depth range of 15–100 m [86,87]. Diet has been reported as primarily benthic and planktonic invertebrates as well as small fishes, though ontogenetic dietary shifts over the lifespan of the fish may also depend on habitat and prey availability. *Pinjalo* snapper have been reported to reach a maximum length of 80 cm [88,89] but are listed as a minor commercial species and harmless in terms of human threat on FishBase.org and related databases. However, many other snappers from the family Lutjanidae have been associated with ciguatera and the noted habitat and feeding patterns fit with the detection of CTXs and ciguatoxicity in this species. Previous reports of ciguatera in the west Indian Ocean include over 30 different fish species, the majority of which were large predators such as groupers [64] and other snappers [33]. To our knowledge, however, this study reports the first confirmed occurrence of ciguatera in *P. pinjalo*.

### 3.3. Testing Methods

For reasons relating to both the absence of commercially available reference standards for an appropriate range of CTX analogues and the complexities associated with developing fit-for-purpose detection methods for ciguatera, there are no formal fully validated methods available for testing implicated fish samples [43,44,90,91]. In addition, complications relate to the low concentrations of toxins present in fish tissues and to the fact that P-, C- and potentially I-CTX variants are structurally different so that methods developed to detect one may not be applicable to another [43,44]. Testing for CTXs traditionally involved the use of live animal (mouse) bioassays, which give a measure of total toxicity [14,92,93,94], but with limited sensitivity, specificity, and ethical concerns. Biomolecular methods such as those utilizing antibodies, the measurement of cytotoxicity through in vitro assays in clonal mammalian neuronal cell lines, radioligand binding receptor assays utilizing tritiated brevetoxin, and the detection of the CTXs themselves through application of chemical detection methods are other approaches which have received wide usage in more recent years [3,10,17,22,32,38,67,68,69,95,96,97,98,99,100,101,102]. Out of the biomolecular approaches, the sodium channel dependent cell viability assay utilizing murine neuroblastoma cells (N2a) in a MTT assay (N2a-MTT or N2a-CBA) is currently the most widely applied, given its sensitivity and applicability to high throughput screening for CTX-like activity whilst also providing an estimate of composite toxicity [10,32,103,104,105]. Whilst a range of chemical detection methods have been reported, the use of liquid chromatography with tandem mass spectrometry (LC-MS/MS) and high-resolution mass spectrometry (LC-HRMS) are the most applicable to detection of low concentrations of toxins [13,23,33,43,44,45,46,47,63,70,85,99,102,106,107,108]. Therefore, for this assessment, two approaches were taken. Fish tissue samples were subjected to both N2a-MTT cytotoxicity assessment for the presence of sodium channel activating toxins as well as LC-HRMS analysis for the confirmation of CTXs. This tiered approach is the current accepted method for regulatory analysis of CTX in fish in the United States, where ciguatera poisonings occur with frequency in Florida, Puerto Rico, and the US Virgin Islands [102,109,110].

### 3.4. Fish Toxicity

Assessment of replicate extracts using the N2a-MTT cell proliferation assay confirmed the presence of sodium channel specific activity in sub-samples taken from the *P. pinjalo* steak implicated in the UK ciguatera poisonings. At the levels detected, human illness would be expected following the consumption of this fish; however, since this fish was sourced from the Indian Ocean, caution should be applied, and the quantification can only be used as a qualitative metric. It should also be noted that the toxicity equivalence between CTX-3C and CTX-1B is also yet to be verified, but having the CTX-3C standard was the only choice available. Since no quantified Caribbean or Indian Ocean CTX standards were available commercially at the time of this study, a C-CTX fish reference material that was prepared at the Dauphin Island Sea Lab (see methods for preparation and testing) was used for qualitative comparison. Ciguatoxicity observed by N2a-MTT assay in a fish matrix containing C-CTX-1 and -2 was consistent with both CTX-3C and the implicated fish sample in this study. It should be noted, however, that N2a-MTT data alone cannot confirm the structural identity or final concentration of any or all CTX congeners that may be present in the implicated sample. However, this confirms that sodium channel specific activity using a standardized method is consistent between a commercially available CTX-3C standard, a reference material confirmed to contain C-CTX-1 and -2 (by LC-MS/MS and LC-HRMS), and the implicated UK sample.

### 3.5. UHPLC-HRMS Analysis

Without quantified and certified standards, the assessment of CTXs in the study was conducted based on known molecular masses, mass spectrometric characteristics and cross verification against other poisoning tissue extracts. Chemical detection using UHPLC-HRMS was used to confirm the presence of chromatographic peaks indicative of Pacific, Caribbean and/or Indian CTXs using evidence from prior studies [33,44,57,63,85,102]. The absence of PSP toxins, or TTX confirmed that the toxicity and illness did not originate from these hydrophilic shellfish biotoxins.

High-resolution MS has previously provided accurate mass ion chromatograms to evidence the presence of CTXs from multiple global regions [45,57,63,77,111,112]. In our mass spectrometer, full-scan UHPLC-HRMS analysis showed the presence of chromatographic peaks with accurate masses with less than 2 ppm mass error, indicative of tri- di- and mono-hydroxylated CTX-3C congeners. Additional evidence was obtained for the presence of 2,3,51-trihydroxy-CTX-3C and 2,3-dihydroxy-CTX-3C through oxidative cleavage using periodate, with UHPLC-HRMS showing expected oxidation products of both compounds, as described previously for confirmation of C-CTX-1, 2, 3, and 4 in fish tissues [77]. Consequently, the 51-hydroxy-CTX-3C could not be confirmed using both approaches. Confirmation of 2,3-51-tryhydroxy-CTX-3C and 2,3-dihydroxy-CTX-3C therefore concurs with the findings of [57] who reported these analogues, in addition to 51-hydroxy-CTX-3C, M-*seco*-CTX-3C, 2-hydroxy-CTX-3C and CTX-3C in samples of red snapper (*L. bohar*) imported into Germany from the western Pacific Ocean and causing a public health concern in 2017 [56]. Such samples were also associated with incidents of human CP, with N2a cytotoxicity results confirming toxicity [55,56,57]. Other groups reporting similar results also included examples of the same hydroxylated CTX-3C congeners in a range of fish species, including *L. bohar* from the tropical western Pacific region [25,70,71,85].

Overall, this study provides further evidence for the presence of hydroxylated CTX-3C congeners in snapper fillets imported from the Indian Ocean/western Pacific region, evidencing multiple structural toxin types from the region including both P- and I/C-CTXs. The I-CTXs initially reported by [33,34] are proposed to be different congeners from C-CTXs based on subtle changes in clinical effects following I.P injection, differing product ion ratios in the mass spectrometer, and earlier elution characteristics for I-CTX following preparative-scale chromatographic purification. It is not known, however, whether these subtle differences result from matrix-related effects or do indeed indicate the I-CTXs to be structurally distinct from the previously reported C-CTX congeners. Our data in combination with prior studies of CTXs conducted in fish collected from the Indian Ocean have reported P-CTX congeners as the detectable CTXs responsible for Europe-wide human health impacts as a consequence of Indian Ocean fish imports. Going forward, purification and isolation of these toxins will be needed to fully elucidate the chemical structures of all analogues potentially present in these tissues, which will be pursued in future studies.

### 3.6. Symptoms and Overall Assessment

Symptoms experienced by human victims of ciguatera are numerous, varied, and complex. Following activation of the voltage-gated sodium channels in cell membranes, increasing sodium ion permeability, the consequent depolarization of the nerve cells results in a poisoning response. Ciguatoxins cause a range of gastrointestinal, cardiovascular and neurological symptoms, generally with a low mortality rate [113], but which can last between several days and several months [10]. As such, many of the ciguatera symptoms differ markedly in comparison to other marine-borne natural toxin poisonings such as Paralytic, Diarrhetic and Amnesic Shellfish Poisoning [114], although some such as the gastrointestinally related are shared [10]. A further complication results from the variability in clinical features depending on the geographical source of the fish consumed [10,64,115,116], though there is significant individual variability reported between vectors, and between patients. Ciguatera diagnosis is generally performed by combining evidence for consumption of fish species implicated or associated with the potential presence of CTXs, scientific data demonstrating the presence of toxins and/or ciguatoxicity, and the presentation of acute clinical symptoms indicative of the syndrome [117]. A universal case definition has been suggested for identification of ciguatera, which is applicable to the different symptoms experienced throughout the world, depending on the geographical source of the implicated fish [10]. These include clinical criteria, i.e., the patient has consumed marine reef fish previously associated with ciguatera, and reports neurological symptoms which may include any combination of paresthesia, dysesthesia, pruritus, allodynia, myalgia, arthralgia, dizziness and even painful ejaculation [10,24,32,118] with onset generally up to 24 h after fish consumption, gastrointestinal symptoms (nausea, vomiting, diarrhea), usually preceding the neurological symptoms between 1 and 12 h after fish consumption. There is also the possibility of cardiovascular symptoms such as hypotension and bradycardia. In addition to the symptoms, classification requires the laboratory confirmation of CTXs in implicated fish meal remnants. While necessary for case confirmation, the ability to obtain a meal remnant for testing is often challenging and this requirement could result in a large underestimation of cases.

In this study, all three patients exhibited gastrointestinal symptoms with vomiting and/or diarrhea within six hours of consuming the fish. Patients 1 and 2 both reported neurological symptoms such as myalgia and paresthesia including skin pain, intense pruritus, numbness, and pins and needles. Patient 3, in addition to gastrointestinal symptoms, experienced pain in the shin bone of one leg as well as reduced strength. Neuropsychological or cardiac symptoms were not reported by any of the patients in this study. As such, given the occurrence of both gastrointestinal and neurological symptoms, all three patients met the clinical criteria for ciguatera diagnosis. Taking the shared medical symptoms, and the known food consumption patterns, together with the known potential for ciguatera poisoning to occur in piscivorous, reef-associated Lutjanids, the subsequent evidence for CTX-like activity and confirmed CTXs in the same fish samples by UHPLC-HRMS provides the evidence for this fish to be the cause of human illness in this event. With two or more cases epidemiologically related, this provides the evidence needed (based on [10]) for confirmation of the first ciguatera poisonings in the UK. Unfortunately, no long-term follow-up with the three patients was carried out by the medical practitioners involved, so post-assessment of disease chronology was not possible.

### 3.7. Legislation and Health Protection

In terms of the protection of human consumers of fish, EU regulations [39,119] state that checks must take place to ensure that fishery products containing biotoxins such as ciguatera or other toxins dangerous to human health are not available in the market. The same legislation also states that poisonous fish from the specific families *Tetraodontidae* (pufferfish), *Molidae* (molas or ocean sunfish), *Diodontidae* (Porcupine fish/globefish/blowfish), also specifically mentioning the genus *Canthigasteridae* (member of the *Tetraodontidae* family) are not to be available in the market, although these are mentioned due to the specific link between these fish and the likely presence of pufferfish poisoning toxins, tetrodotoxin. Currently, no routine CTX testing is undertaken within the UK or US on imported fish products. In parts of the world most affected by ciguatera, some guidance does exist, relating to the purchase and/or consumption of certain species and/or the prevention of fishing certain species above specific weight limits [120]. In regions belonging to France and the US, neurotoxicity screening and LC-MS/MS confirmation has also been undertaken in a post hoc setting (i.e., after poisonings have occurred), but to our knowledge, there are no routine pre-surveillance mechanisms in place for the prevention of ciguatera in local populations or in imported products [121]. Until reliable, validated, and cost-effective CTX-testing methods can be implemented into routine monitoring programs, the most common approach for mitigating ciguatera is the avoidance of reef fish most commonly associated with illness [24] and more generally, avoidance of reef fish harvested from endemic or emerging high-risk regions [120].

In this study, given the clinical symptoms, the toxicity results and the confirmed fish species implicated in the poisonings, the UK authorities concluded that fish from the same batch should not be released for retail sale. Consequently, the recalled products were not released for further sale and samples provided for further study to enable enhanced levels of future public health protection. In conclusion, we report here for the first time the confirmed intoxication in the UK from ciguatera poisoning as a result of toxic carnivorous reef fish being imported into the country and affecting the local population. We therefore highlight the importance of monitoring for the presence of these toxins, and the need to develop risk management strategies for protecting the human population from highly contaminated ciguateric fish.

## 4. Materials and Methods

### 4.1. Fish Speciation

Genomic DNA was extracted from fish samples using an automated system. Briefly, 0.1 g of fish tissue was homogenised in 900 µL of G2 buffer (Qiagen, Manchester, UK) containing 10 µL proteinase K. The sample was incubated at 56 °C for 3 h and the DNA was then extracted from 50 µL of the digest using the DNA tissue mini kit (Qiagen, Manchester, UK) and the EZ-1xl biorobot, as per the manufacturer’s instructions. The nucleic acid was eluted in a 60 µL volume. The primers used in the analysis were modifications of those already published [73,74,75]. A cocktail of 4 primers, Fish COI For1 (AACCAACCACAAAGACATTGGCAC), Fish COI For2 (GACTAATCATAAAGATATCGGCAC), Fish COI Rev1 (TTCAGGGTGACCGAAGAATCAGAA) Fish COI Rev2 (CTCAGGGTGTCCGAARAAYCARAA) was used to amplify a partial COI sequence for confirmation of the species by sequence analysis. Amplifications were performed in a 50 μL reaction volume consisting of 1× GoTaq flexi buffer (Promega, Southampton, UK), 2.5 mM MgCl_2_ (Merck, Poole, UK), 1 mM dNTP mix, 50 pmol of the forward and reverse primers, 1.25 units of GoTaq DNA Polymerase (Promega, Southampton, UK) and 2.5 µL of the purified DNA template (SOP 2007). The reaction mix was overlaid with mineral oil and after an initial denaturing step (5 min at 95 °C), was subjected to 40 temperature cycles (1 min at 95 °C, 1 min at 60 °C and 1 min at 72 °C) in a Peltier PTC-225 thermal cycler (MJ Research, Braintree, UK) followed by a final extension step of 10 min at 72 °C. The PCR was performed in duplicate. PCR products were resolved on a 2% agarose gel containing ethidium bromide by electrophoresis and visualised under UV light. PCR products generated using the COI primers cocktail were extracted and purified by ethanol precipitation using the freeze and squeeze method and both DNA strands of the amplicon were sequenced using the ABI PRISM BigDye terminator cycle sequencing system (Thermo Fisher Scientific, Hemmel Hempstead, UK) and the primers used in the initial amplification. Sequencing reactions were analyzed on an ABI 3500 xl genetic analyzer (Thermo Fisher Scientific, Hemmel Hempstead, UK). A consensus sequence (with primer derived sequences removed) was determined using Sequencher version 5.4 software (Gene Codes Corporation, Ann Arbor, MI, USA) and the origin of the amplicon sequence identified using the Basic Local Alignment Search Tool (BLAST) facility available at the National Centre for Biotechnology Information (NCBI, Bethesda, MD, USA). Species-level identification was confirmed based on % identity to voucher sequences of *P. pinjalo* and sequences deposited on Genbank (NCBI, Bethesda, MD, USA).

### 4.2. Chemicals

All chemicals and reagents used for UHPLC-HRMS analysis were LC-MS-grade (Merck, Poole, UK), and all others were HPLC-grade (Merck, Poole, UK) or equivalent. All cell culture reagents, supplements, fetal bovine serum, and media were of the highest cell culture grade available. Mouse neuroblastoma cell lines, Neuro-2a (ATCC^®^ CCL131™), were purchased from the American Type Culture Collection—ATCC (Manassas, VA, USA) and new lines generated through ouabain and veratrine pre-treatment and desensitization to improve detection limits in subsequent assay use as previously described [13]. A CTX-3C standard used for UHPLC-HRMS optimization was obtained from FujiFilm Wako Pure Chemical Corporation (Osaka, Japan).

CTX reference materials for the N2a-MTT assay were prepared at the Dauphin Island Sea Lab from *Scomberomorus cavalla* (King mackerel) and *Sphyraena barracuda* (Great barracuda) collected from long term monitoring sites on the Southeast coast of St. Thomas, US. Virgin Islands in 2014–2016 (permit STT-015-11, Dept. Planning and Natural Resources, Virgin Islands) as previously described [77].

### 4.3. Fish Samples

The frozen fish steak linked to the ciguatera incident had been kept frozen until cooking and consumption. The package contained four steaks, each around 10 mm in thickness, all cut across the full width of the fish, thereby containing a cross section of edible flesh, together with the outer skin and a segment of backbone. The one remaining fish steak from the purchaser was shipped to Cefas frozen in a temperature controlled cool box. Once received, the tissue was allowed to thaw to room temperature, cut into small chunks with scissors and blended using a high-speed Waring blender. Stringy fragments of skin were removed, and thorough homogenization achieved from the remaining edible flesh. The homogenized tissue sample was divided into sub-samples and re-frozen (−80 °C) prior to subsequent analysis. Red snapper (*L. bohar* from Vietnam) samples were also received from Germany following human poisonings [57,122] and used for cross referencing CTX-3C congener detection (Supplementary Materials).

### 4.4. Extraction Procedure for Cytotoxicity (N2a-MTT) Assay

Fish flesh samples (2 g) were each extracted with 4 mL acetone twice using a bead mill homogenizer (Beadruptor-24, Omni, Kennesaw, GA, USA) with 2.8 mm ceramic beads. Tissue disruption was conducted during acetone extraction in two cycles of 30 s duration at a speed of 5 m/s. Samples were centrifuged between extractions and supernatants pooled into glass test tubes stored at −80 °C for at least 4 h to precipitate proteins. Clarified samples were then taken to dryness under purified N_2_(*g*), resuspended in 2 mL 90% aqueous methanol and defatted twice with 4 mL hexane before being dried, resuspended in 4 mL CHCl_2_ and partitioned with equal volumes of water twice. Samples were then dried under N_2_(*g*) and further purified by solid phase extraction (SPE) prior to N2a assay to reduce matrix effects. Agilent (Manchester, UK) Bond Elut SI (1 g/6 mL) cartridges were first conditioned using 6 mL 95% aqueous MeOH, followed by 12 mL MeOH then 12 mL CHCl_3_. The 1 mL CHCl_3_ sample was added to the top of cartridge, washed using 12 mL CHCl_3_ and the toxins were eluted using 12 mL of 10% MeOH: 90% CHCl_3_. SPE eluants were evaporated to dryness before resuspending in 0.5 mL MeOH.

### 4.5. N2a-MTT Cell Proliferation Assay

Cleaned fish tissue extracts were examined for ciguatoxicity using the sodium channel-specific MTT assay with mouse neuroblastoma cells (N2a-MTT) to evaluate changes in cell viability following various treatments and controls. Clonal cells were purchased from the supplier (American Type Culture Collection, ATCC, Manassas, VA, USA) and grown to 90% confluency until cell viability was >95% and measured growth rates were stable across 10 passages. Once stable, modified cell lines were prepared via pretreatment with ouabain and veratrine as previously described ([12,13]; Supplementary Materials). All cell lines were authenticated by sequencing to verify species origin and were verified mycoplasma-free before and after use since this type of contamination is difficult to pick up by standard microscopy but can affect cell sensitivity, growth, and downstream assay reproducibility. Assay cell stocks were also limited to maintenance for 2–3 months (i.e., no more than 90 passages) to reduce mutations caused by extended culture. Likewise, antibiotics were not used at any point in the N2a maintenance or cell-based assays to reduce the uncertainty associated with antibiotic induction of xenobiotic gene expression (e.g., [123]), possible mitochondrial dysfunction (e.g., [124]), antibiotic-induced oxidative stress, and other changes to cell growth and biochemistry that could result in anomalous results.

Prior to N2a-MTT assay, replicate flasks of at least two distinct passages of modified N2a cells (DISL-N2a-101) were grown in 175 cm^2^ culture flasks until 80–90% confluent. Cells were harvested in trypsin-EDTA (0.025%), deactivated and washed, and then counted and evaluated to meet quality control criteria (cell viability of population > 95%). Cells were then seeded into cell culture treated 96-well polystyrene plates (Celltreat; Pepperell, MA, USA) at 30,000 cells/well. Dose response experiments were conducted after approx. 18 h, once cells reached 50–60% confluency in sample wells. Briefly, ouabain octahydrate (2.5 mM), veratrine hydrochloride (0.25 mM) (OV), and/or sterile phosphate-buffered saline (PBS; pH 7.4) were added to the sample wells containing DISL-N2a-101 cells. The snapper extracts, controls, and reference materials were reconstituted in 5% FBS-RPMI media at a maximum of 20 mg tissue equivalent (TE) dose with a 2.0% MeOH vehicle and serially diluted (20–0.16 mg TE). Standard curves were generated with CTX-3C with a starting dose of 25 pg and 2× serially diluted to provide at least 8 points across the curve. Samples and standards were then applied to the sensitized (dosed with OV) and non-sensitized cells (dosed with PBS) in triplicate across two distinct cell passages. Additional quality assurance and quality controls were incorporated into the workflow to ensure high quality, reproducible data as previously described [13]. Dosed cells were incubated in a humidified environment for 18 h at 37 °C with 5% CO_2_. After incubation, thiazolyl blue tetrazolium bromide (MTT) was added to plates and incubated at 37 °C for 25–30 min to produce a formazan product in live cells remaining in the wells. Dimethyl sulfoxide (DMSO) was then added to dissolve the formazan deposits and develop the final colorimetric endpoint. Plates were then read on a 96-well spectrophotometer with Magellan software version 7 (TECAN Sunrise; Morrisville, NC, USA) at a measurement wavelength of 570 nm.

Dose–response curves (8 doses) were constructed following normalization of the data to % OV control as is deemed best practice for MTT based assays and performing a logarithmic transformation of the dose. Full curves from the standards and fish reference materials were compared to the snapper extracts and analyzed by a logistic four parameter nonlinear regression with variable slope (GraphPad Prism, v7, GraphPad Software, Boston, MA, USA). Measurements between replicates with a percent coefficient of variance less than 10% were considered acceptable. The inhibitory concentration at which 50% of the cells remained viable (IC_50_) were compared with the available commercial standard CTX-3C to provide an estimate in nanograms per gram CTX-3C equivalents. A C-CTX reference fish was also analyzed in parallel to assess similarities and differences between toxin classes; however, a quantified standard was not available for the determination of concentrations in the extracts as C-CTX equivalents.

### 4.6. Full Scan UHPLC-HRMS

Fish flesh samples (10 g) were extracted following an approach based on [125] with 30 mL MeOH:water (3:2 *v/v*) using an UltraTurrax homogenizer (IKA, Oxford, UK). Tissue disruption was conducted in two cycles of 60 s duration at a speed of 5 m/s. Following homogenization, samples were then boiled for 10 min. Cooled samples were then centrifuged (10 min, 4500× *g*) with the clear supernatant transferred into a clean 50 mL tube and left in the freezer overnight to precipitate insoluble material. The next day, liquid–liquid partitioning was conducted by first adding 20 mL of dichloromethane (DCM), the contents shaken and allowed to separate before transferring the bottom DCM layer into a round-bottom flask. The DCM partitioning was repeated two more times, after which, both times, the DCM layer was added to the same round-bottom flask. The DCM fraction was subsequently rotary evaporated to dryness to ensure any traces of water were removed, before being re-dissolved in 5 mL DCM and transferred to a smaller glass tube. The solution was then re-evaporated using a Genevac miVac Quatro centrifugal concentrator (Fisher Scientific, Loughborough, UK) before re-dissolving in 1.0 mL DCM ready for solid phase extraction (SPE) clean-up.

Normal-phase SPE was conducted using JT Baker Bakerbond 1 g/6 mL Florisil cartridges (VWR, Lutterworth, UK) initially conditioned using 10 mL MeOH/H_2_O (95:5 *v*/*v*), then 10 mL MeOH, prior to 20 mL DCM. The 1 mL sample was added to the SPE, before rinsing with 10 mL DCM. The cleaned-up sample was then collected in 20 mL MeOH/DCM (1:9 *v*/*v*), with the eluant evaporated in the Genevac miVac. The dried residue was subsequently re-dissolved in 1.0 mL MeOH prior to analysis.

UHPLC-HRMS analysis was conducted using a Vanquish UHPLC system coupled to an Orbitrap Exploris 120 high resolution mass spectrometer (Thermo Scientific, Hemel Hempstead, UK) with ciguatoxins separated using a 1.7 µm, 2.1 × 100 mm Waters (Manchester, UK) Acquity UHPLC BEH C18 column in conjunction with a Waters VanGuard BEH C18 1.7 µm 2.1 × 5 mm guard cartridge. The column was held at 40 °C, with samples held in the sample manager at 10 °C. The sample injection volume was 2 µL, and the mobile phase flow rate was 0.40 mL/min, with mobile phases A consisting of 5 mM ammonium formate and 0.1% formic acid in water and B being 100% acetonitrile. The UHPLC gradient was as follows: 58% B starting conditions, holding for 1 min then rising to 100% B at 10 min and holding for 1 further min, before dropping back to 58% B at 11.5 min and equilibrating until the end of the run at 15 min.

The developed HRMS method utilized the following source conditions after optimization using the Wako CTX-3C standard: H-ESI source type, static spray voltage, 4000 V positive ion voltage, sheath gas, aux gas and sweep gas at 50, 10 and 1 arbitrary units, respectively, 325 °C ion transfer tube, 225 °C vaporizer temperature. EASY-IC™ internal mass calibration was used during the analysis, with the LC flow diverted to waste until 0.2 min. Full scan was conducted over *m*/*z* 950–1250 using maximum resolution of 120,000 and an RF lens of 70%, with full scan data interrogated using Thermo Scientific FreeStyle analysis software v1.8 (Thermo Scientific, Hemel Hempstead, UK).

In addition, the fish tissue was analyzed for paralytic shellfish poisoning (PSP) toxins and tetrodotoxins, shellfish toxins which also cause toxicity through their action on sodium channels. Standard, validated methodologies were used for these assays [126,127].

### 4.7. Periodate Oxidation

A 20 µL aliquot of the SPE-cleaned fish extract was subjected to oxidative cleavage of the vicinal diols based on the method of [77]. The reaction was achieved through the addition of 20 µL periodate reagent, prepared freshly from 0.15 M periodic acid, 0.3 M ammonium formate and 0.3 M Na_2_HPO_4_. Control samples were also prepared including equal volumes fish extract and water, plus equal volumes water and periodate reagent. The mixtures were heated to 45 °C for 35 min, before cooling and analysis by LC-HRMS, as above.

### 4.8. Medical Observations

Following the hospitalization, anonymized medical records were provided by two out of the three affected people.

## 5. Conclusions

This study reports the first confirmed occurrence of Ciguatera Poisoning in the UK, following the consumption of fish steaks imported into the country from products harvested in the Indian Ocean. Whilst the fish products were labeled as Red Snapper (*Lutjanus bohar*), DNA barcoding confirmed the species to be the Pinjalo Snapper (*Pinjalo pinjalo*). Three hospitalized patients exhibited gastrointestinal symptoms, with two of these reporting neurological symptoms, all meeting the clinical diagnosis criteria for Ciguatera Poisoning. Toxicity was determined in one of the implicated fish steaks using the N2a-MTT cytotoxicity assay, which revealed quantitative concentration of 1.81 µg CTX-3C equivalents per kg tissue. Whilst no Caribbean or Indian Ocean CTX analogues were detected, high-resolution mass spectrometry confirmed the presence of tri-, di- and mono-hydroxylated CTX-3C congeners to within 2 ppm mass accuracy, with further confirmation obtained through periodate triggered oxidative cleavage and the detection of di- and tri-hydroxy CTX-3C reaction products. Consequently, this study demonstrates the use of multiple detection methods for the confirmation of Ciguatera Poisoning in UK patients following consumption of a mis-labeled fishery product from the Indian Ocean, and the importance of continued surveillance of imports to the UK from high-risk regions.

## Figures and Tables

**Table 1:** Summary of HRMS identification of CTX3C congeners.

CTX3C Congener	Formula	Ion	Theoretical *m/z*	Observed *m/z*	Mass Error (ppm)	Retention Time (min)
2,3,51-trihydroxy-CTX-3C	C_57_H_84_O_19_	[M + H]^+^	1073.5680	1073.5663	−1.58	6.84
2,3,51-trihydroxy-CTX-3C	C_57_H_84_O_19_	[M + Na]^+^	1095.5499	1095.5507	−0.73	6.84
2,3-dihydroxy-CTX-3C	C_57_H_84_O_18_	[M + H]^+^	1057.5730	1057.5747	1.61	7.59
51-hydroxy-CTX-3C	C_57_H_82_O_17_	[M + H]^+^	1039.5625	1039.5602	−2.21	8.61

**Table 2:** Summary of medical reports from three patients in suspected ciguatera poisoning cases.

	Patient 1	Patient 2	Patient 3
Admission date	22 June 2017	22 June 2017	23 June 2017
Discharge date	24 June 2017	24 June 2017	Not recorded
Sex	Female	Female	Female
Year of birth	1996	1972	2003
Presenting complaint	Vomiting, myalgia	Nausea, vomiting, diarrhea, myalgia	Pain in the right shin
Allergies	None known	None known	None known
Co-morbidities	Asthma	Hypertension	None known
post-discharge	Food poisoning: likely/potential ciguatera poisoning	Food poisoning: likely/potential ciguatera poisoning	Ciguatera poisoning: non-specific pains
Additional analyses conducted	Full blood count (FBC), urea and electrolytes (U&E), liver function test (LFT) = normal	FBC, U&E, LFT: No apparent disorder	U&E: Urea 2.3 mmol/L, Creatinine 50 µmol/L, Sodium 142 mEq/L, Potassium 3.4 mmol/L
	Creatinine kinase = normal	Electrocardiogram: No apparent distress	
	C-Reactive Protein 8.7 mg/L = mild elevation		
Procedures	Ward based care	Ward based care	Blood taken for analysis.
	Intravenous fluid infusion	Intravenous fluid infusion	
Post-discharge medications	Paracetamol, codeine	Chlorphenamine, ibuprofen	None
Follow up	No	No	No

## Data Availability

All relevant data is displayed in this manuscript or Supplementary Materials.

## Supplementary Materials

The following supporting information can be downloaded at: https://www.mdpi.com/article/10.3390/md23020067/s1, Supplementary methods for cross-referencing including sample preparation and LC-HRMS analysis. Figure S1—LC−HRMS extracted ion chromatograms of tri-hydroxyCTX3C ([M + H]^+^ *m*/*z* 1073.5680), di-hydroxyCTX3C ([M + H]^+^ *m*/*z* 1057.5730), 51-hydroxyCTX3C ([M + H]^+^ *m*/*z* 1039.5625) and 2/3-hydroxyCTX3C ([M + H]^+^ *m*/*z* 1041.5781) in (A) *L. bohar* tissue extract from an outbreak in Vietnam, provided by the German Federal Institute for Risk Assessment (Spielmeyer et al., 2022; Loeffler et al. 2022) and (B) *Pinjalo pinjalo* tissue extract from the lot of fish material seized from the UK ciguatera outbreak. Chromatograms are normalized to the most abundant peak (*m/z* 1057.5730, RT 11.05 min) in each sample. Figure S2—Reaction products following periodate oxidative cleavage of a) di-hydroxylated CTX-3C and b) tri-hydroxylated CTX-3C
