# Supplementary material for: First Confirmed Occurrence of Ciguatera Poisoning in the UK from Imported Pinjalo Snapper (Pinjalo pinjalo)
Source: Mar Drugs. 2025 Feb 6;23(2):67. doi: 10.3390/md23020067 (PMC11857775; doi:10.3390/md23020067)
Supplement: Supplementary file 1 [file marinedrugs-23-00067-s001.zip › marinedrugs-3426355-supplementary.pdf]

Supplementary Information

Supplementary Methods for cross-referencing

Sample Preparation

Fish tissues were obtained from a bulk shipment of seized material of *Pinjalo pinjalo* from the same shipment of fish implicated in the CFP outbreak. Tissue was homogenized, stored in vacuum sealed packaging and stored at −20 °C. Two extracts of *Lutjanus bohar* collected in Vietnam were provided by the German Federal Institute for Risk Assessment, National Reference Laboratory for Monitoring of Marine Biotoxins (Berlin, Germany) for confirmation of CTX-3C congeners (Spielmeyer et al., 2022; Loeffler et al., 2022).

Fish tissue was extracted as described by Kryuchkov et al. (2020) with minor modifications. In short, 5 g subsamples were extracted with acetone at a ratio of 3 mL/g (15 mL) and homogenized for 2 min with an Omniprep homogenizer, followed by centrifugation at 3400 *g* for 5 min (4 °C). The supernatant was removed and stored at −20 °C overnight. The acetone extract was centrifuged again at 3400 *g* for 5 min (4 °C) to remove precipitated proteins and evaporated to dryness in a vacufuge at 45 °C (Savant SPD2010, SpeedVac Concentrator). Residues were dissolved in 90% MeOH (5 mL) and partitioned with hexane (10 mL). The solution was vortex mixed for 2 min and centrifuged at 3000 *g* for 5 min. The hexane was discarded and the MeOH fraction was evaporated to dryness in the vacufuge. The residue was suspended in water (5 mL) and CH_2Cl_2 (3 mL), vortex-mixed and centrifuged at 260 *g* for 5 min. The organic layer was removed and the aqueous layer extracted two additional times with CH_2Cl_2. The CH_2Cl_2 fractions were pooled and evaporated to dryness under N_2 (40 °C) to remove residual water, resuspended in CH_2Cl_2 (2 mL) and subjected to Silica SPE (200 mg Strata-Si, Phenomenex). The Si SPE was preconditioned with MeOH (3 mL) and CH_2Cl_2 (3 mL). The sample was added and rinsed with CH_2Cl_2 (5 mL) and the samples were eluted in 6 mL 1:9 (MeOH:CH_2Cl_2). The eluate was evaporated under N_2 at 40 °C and dissolved in 200 μL MeOH for analysis (25 g Tissue Equivalence /mL).

LC–HRMS Analysis

The method employed was adapted from Kryuchkov et al. (2020) as described by Mudge et al. (2022, 2023). Analyses were performed using an Agilent 1290 Infinity II LC equipped with a binary pump, temperature controlled autosampler (10 °C) and temperature-controlled column compartment (40 °C) (Agilent Technologies, Mississauga, ON, Canada) coupled to a Q Exactive HF Orbitrap mass spectrometer (Thermo Fischer Scientific, Waltham, MA, USA) with a heated electrospray ionization probe (HESI-II). Chromatographic separation used a Kinetex F5 UHPLC

column (100 × 2.1 mm, 1.7 μm) with gradient elution and mobile phases composed of 0.1% formic acid in H_2O (A) and 0.1% formic acid in MeCN (B). The gradient (0.3 mL/min) was: 0–18 min, 30–60% B; 18–18.1 min, 60–99% B; 18.1–22 min, 99% B; followed by an 8 min re-equilibration at 30% B. The injection volume was 5.0 μL.

Full-scan acquisition was performed in positive ionization mode with a mass range of *m*/*z* 1000–1250. The spray voltage of the source was 4500 V, with a capillary temperature of 340 °C, and the sheath and auxiliary gas were set at 40 and 10, respectively. The probe heater temperature was set at 150 °C and the S-Lens RF level was set to 100. The mass resolution setting was 120,000 with an AGC target of 3×10^6 and a maximum injection time of 250 ms per scan. Extracted ion chromatograms were used to monitor each ciguatoxin with a mass tolerance of ±5 ppm of the most abundant ion.

Supplementary Figures

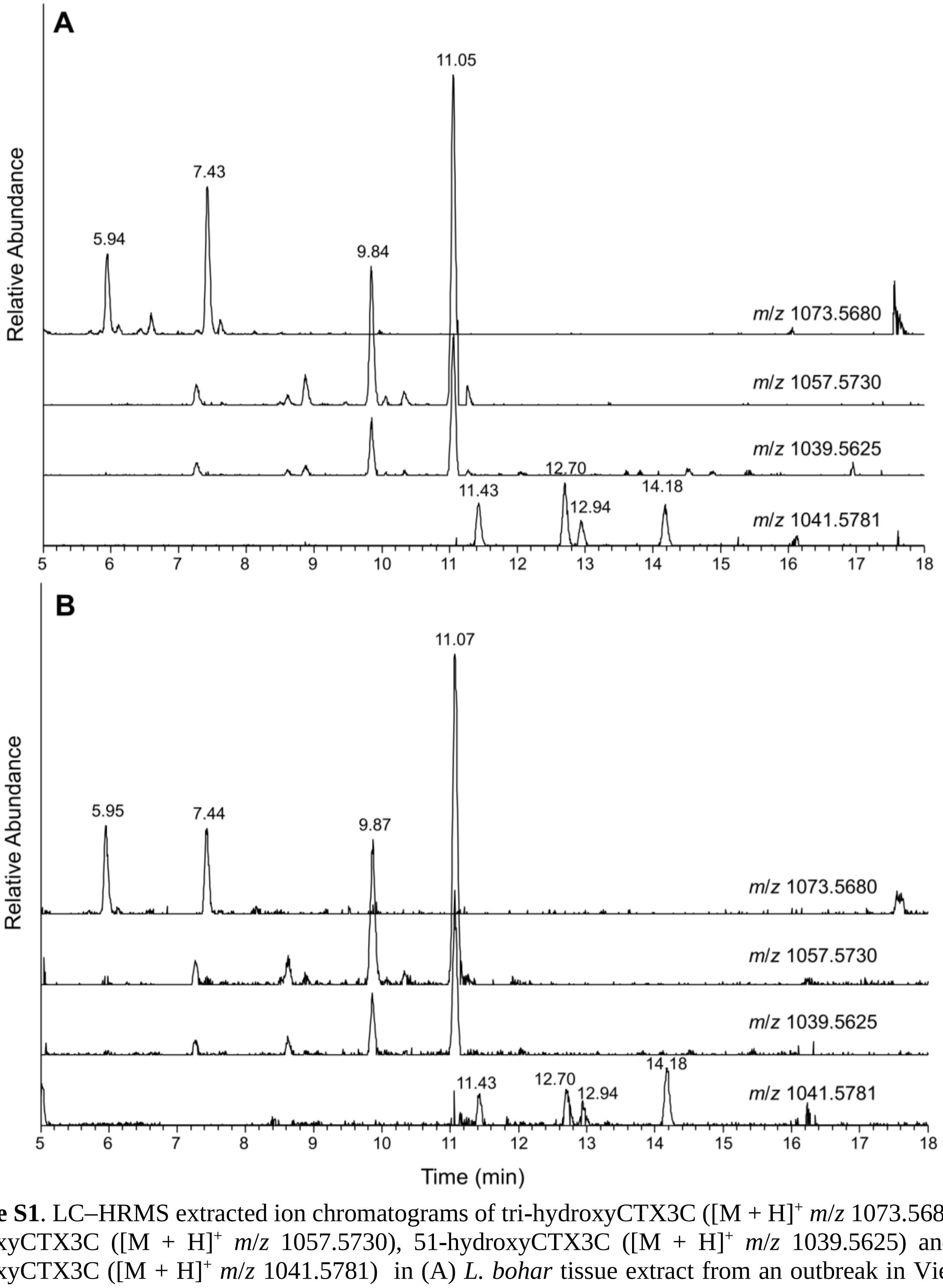

Figure S1. LC–HRMS extracted ion chromatograms of tri-hydroxyCTX3C ($[M + H]^+$ *m/z* 1073.5680), di-hydroxyCTX3C ($[M + H]^+$ *m/z* 1057.5730), 51-hydroxyCTX3C ($[M + H]^+$ *m/z* 1039.5625) and 2/3-hydroxyCTX3C ($[M + H]^+$ *m/z* 1041.5781) in (A) *L. bohar* tissue extract from an outbreak in Vietnam, provided by the German Federal Institute for Risk Assessment (Spielmeyer et al., 2022; Loeffler et al. 2022) and (B) *Pinjalo pinjalo* tissue extract from the lot of fish material seized from the UK ciguatera outbreak. Chromatograms are normalized to the most abundant peak (*m/z* 1057.5730, RT 11.05 min) in each sample.

Figure S2. Reaction products following periodate oxidative cleavage of a) di-hydroxylated CTX-3C and b) tri-hydroxylated CTX-3C

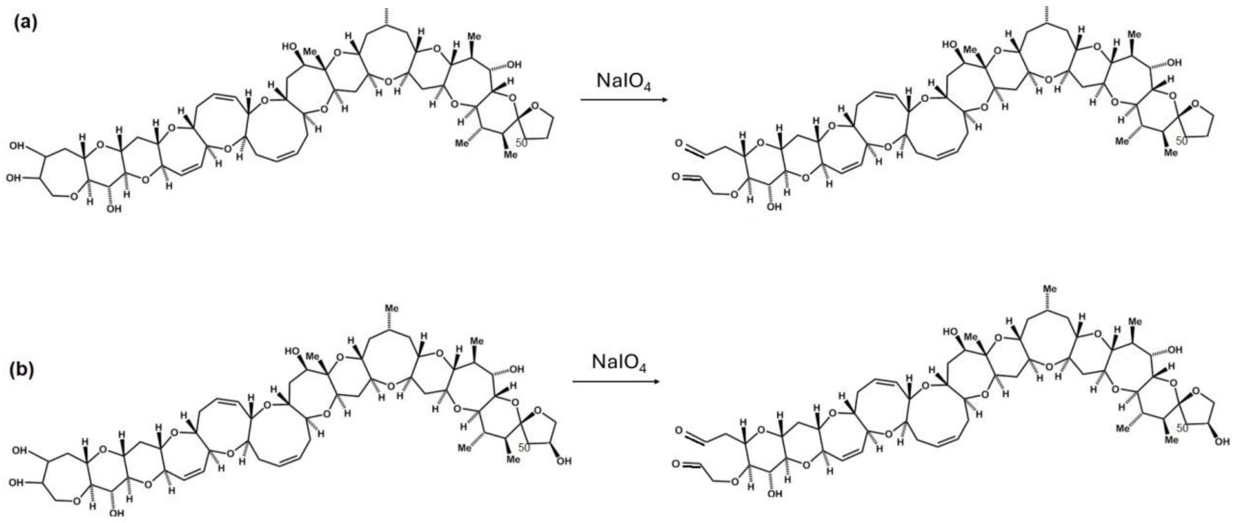

References

1. Spielmeyer, A.; Loeffler, C. R.; Kappenstein, O., Identical ciguatoxin-3C group profiles in *Lutjanus bohar* from the Pacific and Indian Oceans - indicating the need to re-evaluate geographical CTX classifications. *Front. Mar. Sci.* **2022,** 9.
2. Loeffler, C. R.; Spielmeyer, A.; Friedemann, M.; Kapp, K.; Schwank, U.; Kappenstein, O.; Bodi, D., Food safety risk in Germany from mislabeled imported fish: ciguatera outbreak trace-back, toxin elucidation, and public health implications. *Front. Mar. Sci.* **2022,** 9.
3. Kryuchkov, F.; Robertson, A.; Miles, C. O.; Mudge, E. M.; Uhlig, S., LC–HRMS and chemical derivatization strategies for the structure elucidation of Caribbean ciguatoxins: Identification of C-CTX-3 and -4. *Mar. Drugs* **2020,** 18, (4), 182.
4. Mudge, E. M.; Robertson, A.; McCarron, P.; Miles, C. O., Selective and efficient capture and release of *vic*-diol-containing Pacific and Caribbean ciguatoxins from fish extracts with a boronate affinity polymer. *J. Agric. Food Chem.* **2022,** 70, (40), 12946–12952.
5. Mudge, E. M.; Miles, C. O.; Ivanova, L.; Uhlig, S.; James, K. S.; Erdner, D. L.; Fæste, C. K.; McCarron, P.; Robertson, A., Algal ciguatoxin identified as source of ciguatera poisoning in the Caribbean. *Chemosphere* **2023**, 138659.
